# Day case laparoscopic nephrectomy: initial experience

**Published:** 2011-02-25

**Authors:** CP Ilie, CJ Luscombe, I Smith, J Boddy, D Mischianu, A Golash

**Affiliations:** * Department of Urology, Central Military Emergency University Hospital, ‘Carol Davila’ University of Medicine and Pharmacy, BucharestRomania; ** Department of Urology, University Hospital of North Staffordshire, Stoke on TrentUK; *** Department of Anesthesiology, University Hospital of North Staffordshire, Stoke on TrentUK

**Keywords:** day surgery, laparoscopic procedure, Clavien system

## Abstract

**Rationale**: Laparoscopic nephrectomy tends to become the new gold standard surgical technique in a selected population (non–functioning kidney, localised renal cell carcinoma). Day surgery is a popular pathway of care and, procedures of ever–increasing complexity are being considered.

**Objective**: The aim of the study was to report the postoperative complications of day case laparoscopic nephrectomy, according to the Clavien system, and, to assess the feasibility of the procedure performed as a day case.

**Material and Results**: This study included all the patients considered for day case transperitoneal laparoscopic nephrectomy between May 2008 and November 2009. Sixteen consecutive patients were enrolled in this retrospective study.  There were ten procedures on the left hand–side and six on the right hand–side. Age ranges from 22 to 77 years old. Male to female ratio was 9:7. The preoperative diagnosis was non–functioning kidney in 9 cases and kidney tumour in the other 7 cases. All but two patients have been discharged in the same day (87.5%). The readmission rate was of 12.5%. One wheel–chair bonded patient was readmitted four days after the procedure, because of adynamic ileus, and another one three days later because of wound infection. There were two grade I and one grade IV complications (Clavien system). The patient readmitted with grade IV complication, wheel–chair bonded because of cerebral palsy, was not a typical day surgery patient.

**Discussion**: The vast majority of complications were minor and resulted in no residual disability. In our small series, the day case laparoscopic nephrectomy was feasible and safe.

**Abbreviations**: day surgery (DS), laparoscopic nephrectomy (LN), American Society of Anesthesiology (ASA)

## Introduction

Day surgery (DS) is defined as the admission of selected patients to hospital for a planned surgical procedure, returning home on the same day. Day surgery is an important medical advance because it provides benefits to all parts involved: patients receive treatment that is suited for their needs and, in the same time, it allows them to recover in their own home. The risk of hospital acquired infection is reduced and clinicians can provide high quality care, and release inpatient beds for more major cases [1,2].

The first laparoscopic nephrectomy (LN) was performed 20 years ago [3]. The place of this procedure in the urologic armamentarium is well established. So far, to the best of our knowledge, there is no report of laparoscopic nephrectomy performed as day case. 

Eighteen years ago, Clavien et al first published a system in which complications were systematically graded, and that paper is the basis of the Clavien system [4]. In 2004, Dindo et al re–evaluated and modified the criteria to increase its accuracy and applicability [5].

The aim of our study was to report the postoperative complications of day case laparoscopic nephrectomy according to the Clavien system and, to assess the feasibility of the laparoscopic nephrectomy performed as a day case.

## Meth

We retrospectively studied a series of consecutive patients planned for DS LN over a period of 18 months, between May 2008 and November 2009. It is important to emphasize that all patients planned to be performed as DS, followed a standard pathway of care. After the indication for LN was established, patients were selected for DS or inpatient procedure. Initially young, fit and well–motivated patients were considered for DS. After the initial assessment of feasibility, the inclusion criteria were extended to those used for other DS laparoscopic procedures, such as laparoscopic cholecystectomy. There were medical, surgical and social criteria. The medical criteria consist of patients with an ASA (American Society of Anesthesiology) equal or below 2 and with any coexisting medical conditions stable and optimally treated. From the surgical point of view, our selection criteria are related to the size and nature of the renal pathology and hence, to the size of the extraction site and the likelihood of postoperative complications. In the same time, social criteria have been considered: the patient had to be willing to undergo DS; following the procedure, there was a responsible adult/carer/parent able and willing to care for the patient for at least 24 hours, patients/parents had access to a private telephone, the journey home took no longer than one hour and an escort was available to drive or accompany the patient home, in a taxi. Therefore, when all those criteria were met, we proposed a DS procedure to the patients, as an option to an inpatient procedure.

All the patients from this series accepted the DS procedure. A confirmation phone call was made the day prior to admission. Together with the invitations for admission, instructions to fast from twelve midnight for solids and 6.30 am for fluids (approximately two hours before surgery) were also sent. The patients were admitted as any other DS patients, at around 7.30am, in the Day Surgery Ward. They were again informed about the procedure and also, about the need to observe postoperative important signs: increased pain despite analgesia, nausea, increase in temperature and pulse, dizziness and increasing abdominal distension.  The procedure was usually performed as the first case in the morning session. An hour before surgery, each patient received 1600mg of slow release ibuprofen orally. The anaesthetic technique was also standardized: induction with midazolam–alfentanil–propofol and maintenance with sevoflurane in air with controlled ventilation via a tracheal tube. The transperitoneal laparoscopic technique was standard, the modification for DS being the fact that no tube drain or urethral catheter were inserted. Patients received 1 gram of IV paracetamol towards the end of the procedure, and the opioids were avoided. 

In order to minimize the postoperative pain levobupivacaine 0.5% was instilled around the port sites before trocar insertion and at the conclusion of the procedure. 

Postoperatively, in the recovery ward, and then in the day surgery ward, hourly parameters were monitored: pulse rate, blood pressure, temperature, consciousness level and pain scores. Oral fluids and diet were introduced postoperatively as tolerated. Between 6pm and 8pm, all patients were reviewed by a senior urologist and discharged if criteria were met: controlled pain and nausea, stable observation, mobilising and tolerating diet. 

After discharge, the patients had to observe the important signs they were informed about. They were also provided direct access to the urology ward and to the surgical admission unit, by phone, and both wards were informed about the patients. They were provided with a five–day course of ibuprofen and co–codamol to be taken regularly. The District Nurse visited the patients at home to monitor temperature, pulse, blood pressure and wound sites on the evening of surgery, day 1 and 2 postoperatively and thereafter at their discretion. At the first postoperative visit as outpatient, all patients were asked one specific question–‘If they were to have this operation again, would they rather stay overnight or go home the same day?’

Data regarding demographic information, medical comorbidities, preoperative diagnosis, pre and postoperative symptoms, admission as well as discharge date and hour were collected. 

**Figure 1:**
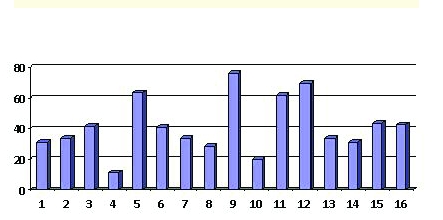
Patients' age

Sixteen patients were offered the option of day surgery during the study period. Whenever offered, each patient's preference was for day surgery. All patients followed a standard pathway of care for DS procedure, as described above. There were seven females and nine males. The procedure was on the left side in ten cases and on the right side in six. The preoperative diagnosis was non–functioning kidney for nine patients and kidney tumour for seven patients. Age distribution is seen in Figure 1. 

## Results

Preoperative symptoms were, for those with non–functioning kidney, pain and urinary tract infections–four patients, pain–four patients, pain and haematuria–one patient; for the patients with kidney tumour: haematuria–one patient; pain–two patients; asymptomatic–four patients. Out of the sixteen patients, eight had medical comorbidities and five had previous scars in different quadrant. The blood loss was minimal for 15 patients and about 150ml in one case.

**Figure 2:**
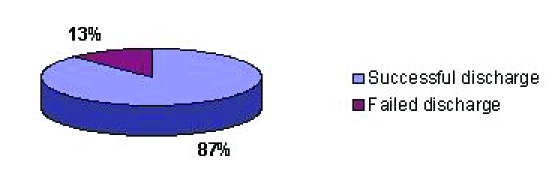
Successful discharge rate

The successful discharge rate was of 87%. Fourteen patients have been successfully discharged and two patients failed discharge in the same day (Figure 2).

They had permission to leave the hospital, next morning respectively, on the second postoperative day. One of them, a 62–year–old male patient, was admitted overnight because he did not pass urine until 22.45. The second one, a 43–year–old female patient, was admitted because of uncontrolled pain and vomiting. This can be considered a grade I postoperative complication according to the Clavien system. 

**Figure 3:**
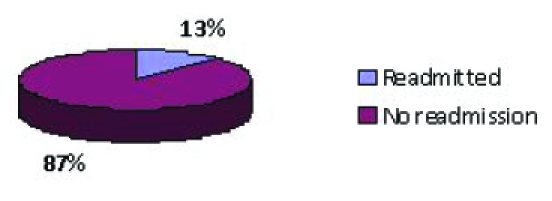
Readmissions

The postoperative follow–up period was of 30 days, looking for readmissions and visits to the surgical assessment unit or the emergency department. Total readmission rate was of 13%. Two patients were readmitted: one patient on the third and the other on the fourth postoperative day (Figure 3). 

The first one, a 31–year–old male patient operated for a left non–functioning kidney, presented to the surgical assessment unit, because of a serous discharge from the epigastric port and right iliac fossa pain. In spite of a non–specific pain, he was admitted overnight for investigation. Blood results were normal and the CT scan of the abdomen was also normal, so the patient was discharged the next day. According to the Clavien system, he had a grade I complication. 

The second readmitted patient was not a typical day case patient. He was wheel chair bonded because of cerebral palsy, had suprapubic cystostomy and also a left staghorn stone with non–functioning kidney. He presented with recurrent urinary tract infections. He was considered for a day case procedure because he had all the necessary support at home, he was very keen and the family insisted on this kind of procedure. He presented on the fourth postoperative day with paralytic ileus and had a slow recovery because of a hospital acquired left lower lobe pneumonia. According to the Clavien system, he had a grade IV complication. If we exclude this patient from our analysis, the readmission rate would be of 6.66%. 

## Discussions

The role of laparoscopic nephrectomy in the urological practice is well established. It is the standard of care for the treatment of most benign diseases in which permanent loss of renal function has occurred and also in tumours and some smaller renal masses not suitable for nephron–sparing surgery [6,7].

Laparoscopic procedures performed as day cases are a natural evolution of the minimal invasive surgery. Many procedures are successfully performed as day case: cholecystectomies, hernia repair, appendectomy, Nissen fundoplication, adrenalectomy and pyeloplasty [8–14].

The new plan of management, for the postoperative care of patients after laparoscopic nephrectomy, was implemented having in mind the patient's safety, because, this is of paramount importance. All safety measures were used, in order to assure a standard of care and to reduce to minimum the possible complications. Every patient, planned for a day case laparoscopic nephrectomy, is following a standard pathway similar to DS cholecystectomy because, this is the most studied day case laparoscopic procedure. 

In our case series, the successful discharge rate was of 87%. Two patients failed to be discharged on the same day. One patient was not able to pass urine until 22.45, when it was too late to be discharged. The second patient had a radical nephrectomy for a kidney tumour, so the extraction scar was bigger and the reason for failing discharge was uncontrolled pain and vomiting. They have been discharged on the first respectively, the second postoperative day. The only data we can compare to is the day case laparoscopic cholecystectomy. Early experience of DS laparoscopic cholecystectomy had a success rate of 56% [15].  Our results are comparable with those published for modern series of laparoscopic cholecystectomies, which are between 86 and 95% [16–19]. 

Total readmission rate was of 13%. Two patients were readmitted. One of them, with cerebral palsy and tetraplegia as comorbidity, was not a typical day case patient, so, if we exclude this patient, we would have a readmission rate of 6.66%. Further analysing the data, we observed that both of them had an uneventful postoperative recovery, so, even as inpatients, they both would have been discharged on the first or the second postoperative day. We do not think that their complications were influenced by the fact that they had a DS procedure. Our readmission rate is not much different from those published in modern series of laparoscopic cholecystectomies (between 1.5 and 8%) [16–20].

This initial study will have to be continued by ones that are more extensive and by formally assessing the patient's satisfaction after this particular procedure. Formal surveys, after a wide range of other day surgery procedures, generally show very high levels of patient satisfaction. In our small case series, all patients answered that they would prefer day surgery when asked, ‘If they were to have this operation again, would they rather stay overnight or go home the same day?’ 

There were two grade I and one grade IV complications according to the Clavien system. The patient presenting the grade IV complication was not a typical day surgery patient. The vast majority of complications were minor and resulted in no residual disability.

In our small series, the day case laparoscopic nephrectomy was feasible and safe. 
